# Monitoring the Formation of Fibrin Clots as Part of the Coagulation Cascade Using Fluorescent Single-Walled Carbon Nanotubes

**DOI:** 10.1021/acsami.3c00828

**Published:** 2023-05-02

**Authors:** Efrat Gerstman, Adi Hendler-Neumark, Verena Wulf, Gili Bisker

**Affiliations:** † Department of Biomedical Engineering, Faculty of Engineering, Tel Aviv University, Tel Aviv 6997801, Israel; ‡ Center for Physics and Chemistry of Living Systems, Tel Aviv University, Tel Aviv 6997801, Israel; § Center for Nanoscience and Nanotechnology, Tel Aviv University, Tel Aviv 6997801, Israel; ∥ Center for Light-Matter Interaction, Tel Aviv University, Tel Aviv 6997801, Israel

**Keywords:** fluorescent nanoparticles, single-walled carbon nanotubes, near-infrared imaging, coagulation cascade, fibrin clots, fibrinogen, thrombin

## Abstract

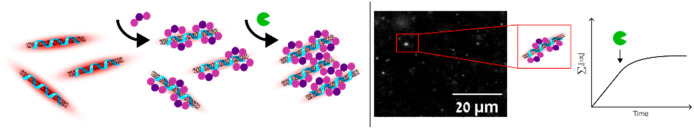

Blood coagulation is a critical defense mechanism against bleeding that results in the conversion of liquid blood into a solid clot through a complicated cascade, which involves multiple clotting factors. One of the final steps in the coagulation pathway is the conversion of fibrinogen to insoluble fibrin mediated by thrombin. Because coagulation disorders can be life-threatening, the development of novel methods for monitoring the coagulation cascade dynamics is of high importance. Here, we use near-infrared (NIR)-fluorescent single-walled carbon nanotubes (SWCNTs) to image and monitor fibrin clotting in real time. Following the binding of fibrinogen to a tailored SWCNT platform, thrombin transforms the fibrinogen into fibrin monomers, which start to polymerize. The SWCNTs are incorporated within the clot and can be clearly visualized in the NIR-fluorescent channel, where the signal-to-noise ratio is improved compared to bright-field imaging in the visible range. Moreover, the diffusion of individual SWCNTs within the fibrin clot gradually slows down after the addition of thrombin, manifesting a coagulation rate that depends on both fibrinogen and thrombin concentrations. Our platform can open new opportunities for coagulation disorder diagnostics and allow for real-time monitoring of the coagulation cascade with a NIR optical signal output in the biological transparency window.

## 1 Introduction

Blood coagulation is an important defense mechanism against bleeding. Rupture of the endothelium allows blood exposure to the extravascular tissue, which initiates the extrinsic coagulation pathway,^1^ while the intrinsic pathway is initiated by coagulation factors (CFs) in the blood.^2^ Both activation pathways lead to a common pathway, which includes the conversion of fibrinogen (CF I) to fibrin (CF Ia), by thrombin (CF IIa).^3^

Fibrinogen, a soluble plasma glycoprotein secreted by the liver, is the most abundant protein in the plasma with normal physiological concentrations of 1.75–4.3 g L^–1^^4^ and the main building block of blood clots. It also acts as an adhesive protein essential for platelet aggregation. Fibrinogen has an elongated structure with three domains, a central E domain and two terminal D domains.^5^ The conversion of fibrinogen to insoluble fibrin involves the proteolytic release of two fibrinopeptides from the amino-terminal ends of the polypeptide chains and it is catalyzed by the enzyme thrombin. The resulting fibrin monomers spontaneously polymerize, forming a fibrin clot.^6^

Thrombin plays a key role in the coagulation cascade because it is responsible for activating fibrinogen and regulating its aggregation. A cascade of CFs initializes the cleavage of prothrombin to thrombin, which then catalyzes the cleavage of fibrinogen and amplifies coagulation through a positive feedback loop.^1,7,8^

The coagulation system is essential for maintaining the balance of blood fluidity and hemostasis. The absence or reduced production of thrombin can result in hemorrhagic diseases like hemophilia,^9,10^ whereas unregulated thrombin production can result in thrombotic occlusion.^11^ Moreover, individuals with congenital fibrinogen deficiency can suffer from uncontrolled bleeding,^5^ whereas elevated levels of fibrinogen increase the risk of thrombosis^12^ and may compromise clot stability.^13^

The prothrombin time (PT) and activated partial thromboplastin time (APTT) are the most common coagulation tests. The PT test involves determination of the time for a clot formation in a plasma sample after initiation of the extrinsic coagulation pathway by the addition of a tissue factor and calcium.^14,15^ The APTT test, on the other hand, measures the clotting time of the intrinsic pathway, which is initiated by the addition of calcium and APTT reagents, containing partial thromboplastin and an intrinsic pathway activator.^16,17^ Because both tests measure the clotting time, they give information only about the end point of the coagulation cascade, where abnormal results require further testing to find the source of the problem.

Two alternative coagulation tests are rotational thromboelastography (ROTEM) and thromboelastography (TEG). These tests are performed on whole blood and measure the viscoelasticity of the blood sample during clot formation in real time. By application of an external shear to mimic blood-flow conditions, the kinetics and strength of the sample are monitored during the clotting process.^18^ While both ROTEM and TEG measure the viscoelastic properties of the blood sample in real time, they only provide bulk properties, without microscopic or spatial information.

Many approaches have been developed to detect fibrinogen or thrombin, such as surface plasmon resonance,^19^ quartz crystal microbalance,^20^ fluorescence,^21,22^ magnetic,^23^ colorimetric,^24^ and electrochemical analyses.^25^ These approaches provide information on the fibrinogen or thrombin concentration but do not provide dynamic information on the coagulation process or the coagulation rate. Other technologies for point-of-care measurements rely on optics, electromechanics, photoacoustics, electrical impedance spectroscopy, and magnetoelasticity for assessing the clotting time; however, their main application is PT and APTT tests.^26^ Therefore, new methods for assessing the coagulation process that can provide dynamic information throughout the cascade would benefit blood coagulation research and advance new diagnostic tools.

Single-walled carbon nanotubes (SWCNTs) can be described as graphene sheets rolled up into long, hollow cylinders of 0.7–3 nm diameter, where different rolling orientations of the graphene sheets lead to different chiralities of the nanotubes.^27^ Each chirality, described by the (*n*,*m*) indexes, has a different diameter and physical, chemical, electronic, and optical properties.^28,29^ Semiconducting SWCNTs fluoresce in the near-infrared (NIR) spectral region, mainly between 900 and 1400 nm, where biological samples are primarily transparent.^30−32^ Further, SWCNTs do not photobleach or blink upon use, provide spatiotemporal information, are stable at room temperature, and have long term biocompatibility *in vivo*.^33−40^ Due to these unique optical properties, SWCNTs become favorable as fluorescent sensors for biomedical applications.^41−51^ The mechanism of SWCNT-based sensors relies on a heteropolymer that is adsorbed onto the SWCNT surface and mediates the binding of a specific target analyte. Analyte binding then modifies the spectral properties of the NIR-fluorescent emission of the SWCNTs providing optical signal readout in real time.^52−54^ This approach has been demonstrated to recognize numerous analytes ranging from small molecules^55−60^ to large proteins and enzymes.^61−68^

Following a high-throughput screening assay, SWCNTs functionalized by dipalmitoylphosphatidylethanolamine (DPPE)–poly(ethylene glycol) (PEG) were discovered as a fibrinogen sensor.^4^ The DPPE–PEG–SWCNT sensor showed a decrease in the SWCNT fluorescence intensity depending on the fibrinogen concentration and could be used to detect and quantify fibrinogen. Fibrinogen was shown to physically bind the SWCNT surface and align along the principal axis of the nanotube, where atomic force microscopy (AFM) imaging revealed the complete binding of the three globular domains of fibrinogen to the SWCNT surface. Moreover, the binding could not be correlated to any nonselective parameters such as the molecular weight, hydrophobicity, or isoelectric point and was thus attributed to structural recognition by the SWCNT corona phase.^4^

**Scheme 1:**
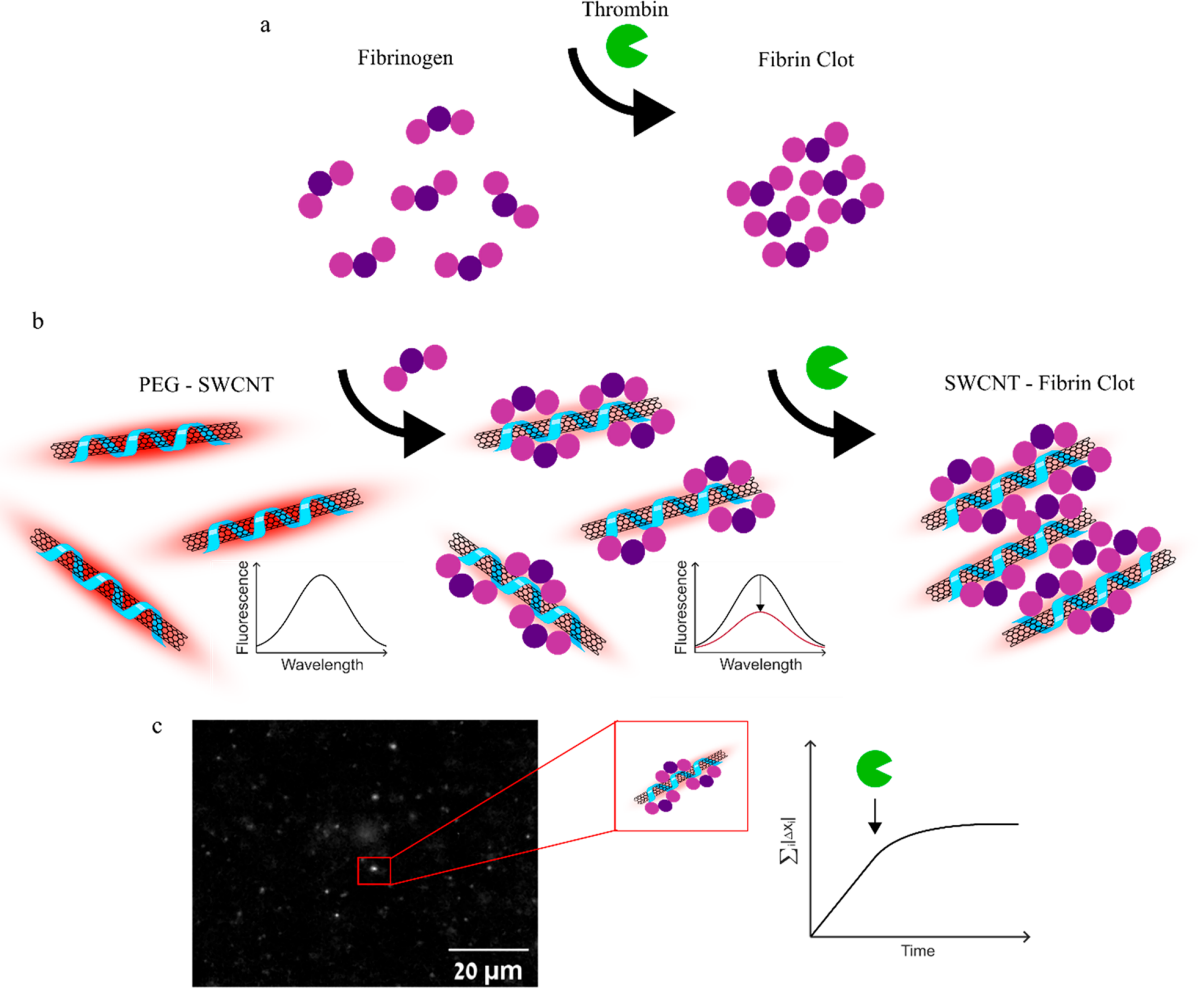
(a) Schematic Illustration of the Fibrin Coagulation Process. Thrombin Catalyzes the Conversion of Fibrinogen to Fibrin, which Polymerizes to Form a Clot. (b) Fibrinogen Binds to DPPE-PEG-SWCNT, Resulting in a Decrease in Fluorescence Intensity. The Addition of Thrombin Leads to Clot Formation Incorporating the DPPE-PEG-SWCNTs. (c) Tracking Individual NIR-Fluorescent DPPE-PEG-SWCNTs during Clot Formation Shows a Gradual Slowing Down of the SWCNT Diffusion Following the Addition of Thrombin

In this work, we show that the DPPE–PEG–SWCNT–fibrinogen sensor can be used as a platform to monitor the catalytic activity of thrombin and visualize the fibrin clot formation, thus providing real-time dynamic information about the coagulation process. We rely on the binding of fibrinogen to DPPE–PEG–SWCNT and the conversion of fibrinogen to fibrin in the presence of thrombin (Scheme 1a). The introduction of thrombin to DPPE–PEG–SWCNT–fibrinogen initiates polymerization of the fibrin monomers, resulting in fibrin clots that encapsulate the SWCNTs (Scheme 1b).^4^ While the binding of fibrinogen to the SWCNT results in a decrease in the fluorescence intensity, the addition of thrombin does not affect the SWCNT fluorescence emission (Scheme 1b). Nevertheless, the SWCNTs are incorporated within the fibrin clots and can be visualized via 2D NIR-fluorescent imaging (Scheme 1c) to provide spatiotemporal information on the clotting process. Moreover, single-SWCNT tracking reveals a gradual slowing down of SWCNT diffusion during the fibrin polymerization process, with both fibrinogen and thrombin concentration-dependent rates. Our platform provides direct visualization of fibrin clotting in real time with a spatiotemporal resolution and can advance the current research and diagnostic tools for the coagulation cascade.

## 2 Results and Discussion

### 2.1 DPPE–PEG–SWCNT Suspension and Functionalization with Fibrinogen

**Figure 1:**
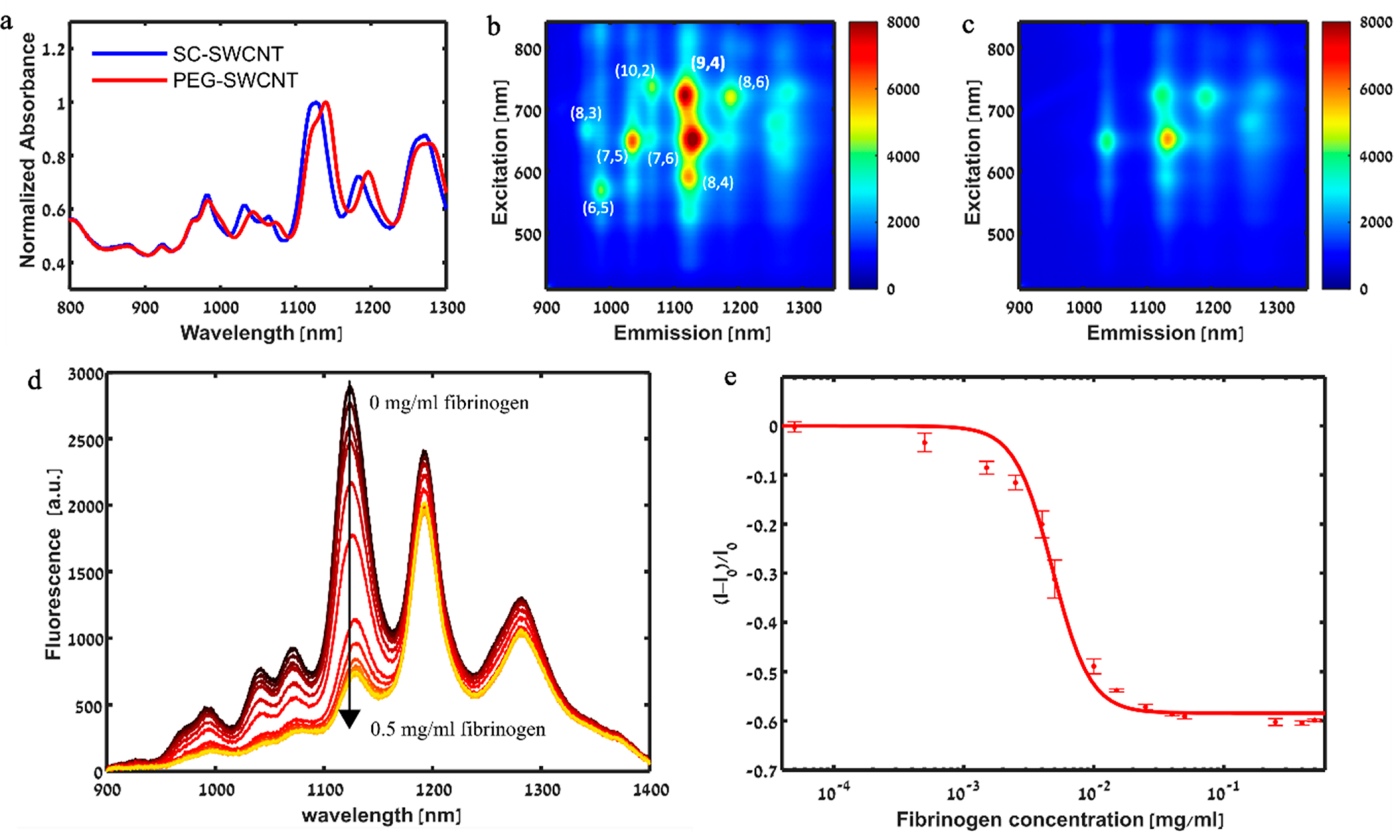
DPPE–PEG–SWCNTs as a platform for fibrinogen absorption. (a) Absorption spectra of a SC–SWCNT suspension (blue) and the red-shifted DPPE–PEG–SWCNT suspension (red). (b) Excitation–emission map of the DPPE–PEG–SWCNTs showing the various nanotube chiralities. (c) Excitation–emission map of the DPPE–PEG–SWCNT solution after the addition of 0.5 mg mL^–1^ fibrinogen. (d) Fluorescence emission spectra of DPPE–PEG–SWCNT under 730 nm laser excitation after incubation with 0, 5 × 10^–5^, 5 × 10^–4^, 1.5 × 10^–3^, 2.5 × 10^–3^, 4 × 10^–3^, 5 × 10^–3^, 1 × 10^–2^, 1.5 × 10^–2^, 2.5 × 10^–2^, 4 × 10^–2^, 5 × 10^–2^, 0.25, 0.4, and 0.5 mg mL^–1^ fibrinogen (black to yellow), showing a concentration-dependent decrease in the fluorescence intensity. (e) Normalized fluorescent response of the (9,4) chirality of the DPPE–PEG–SWCNTs to different concentrations of fibrinogen (●). The solid line represents the fit according to eq 1, as detailed in the Experimental Section.

In order to functionalize SWCNTs with fibrinogen, we used DPPE–PEG-suspended SWCNTs, which were previously shown to specifically bind fibrinogen, even in a serum environment.^4^ SWCNTs were first suspended by sodium cholate (SC), which was then exchanged to DPPE–PEG via dialysis, as is evident from the redshift of the absorption peaks (Figure 1a) and the fluorescence peaks (Figure S1). The DPPE–PEG–SWCNTs showed bright fluorescent peaks of various chiralities in the mixture (Figure 1b). Following the absorption of fibrinogen onto the DPPE–PEG–SWCNTs, an overall decrease in the fluorescence intensity of all of the chiralities was observed (Figure 1c). The DPPE–PEG–SWCNTs concentration-dependent fluorescence intensity response to fibrinogen (Figure 1d) could be calibrated to the fibrinogen concentration, reaching the maximal saturated value at approximately 0.012 mg mL^–1^ (Figure 1e). The ability of fibrinogen to be efficiently adsorbed onto the DPPE–PEG–SWCNTs demonstrates that the SWCNTs are a promising platform to be integrated into the fibrin clots, even at low fibrinogen concentrations, and provide optical signal readout in the NIR range for visualization, localization, and tracking.

### 2.2 Fluorescence Properties of DPPE–PEG–SWCNTs–Fibrinogen in the Presence of Thrombin

**Figure 2:**
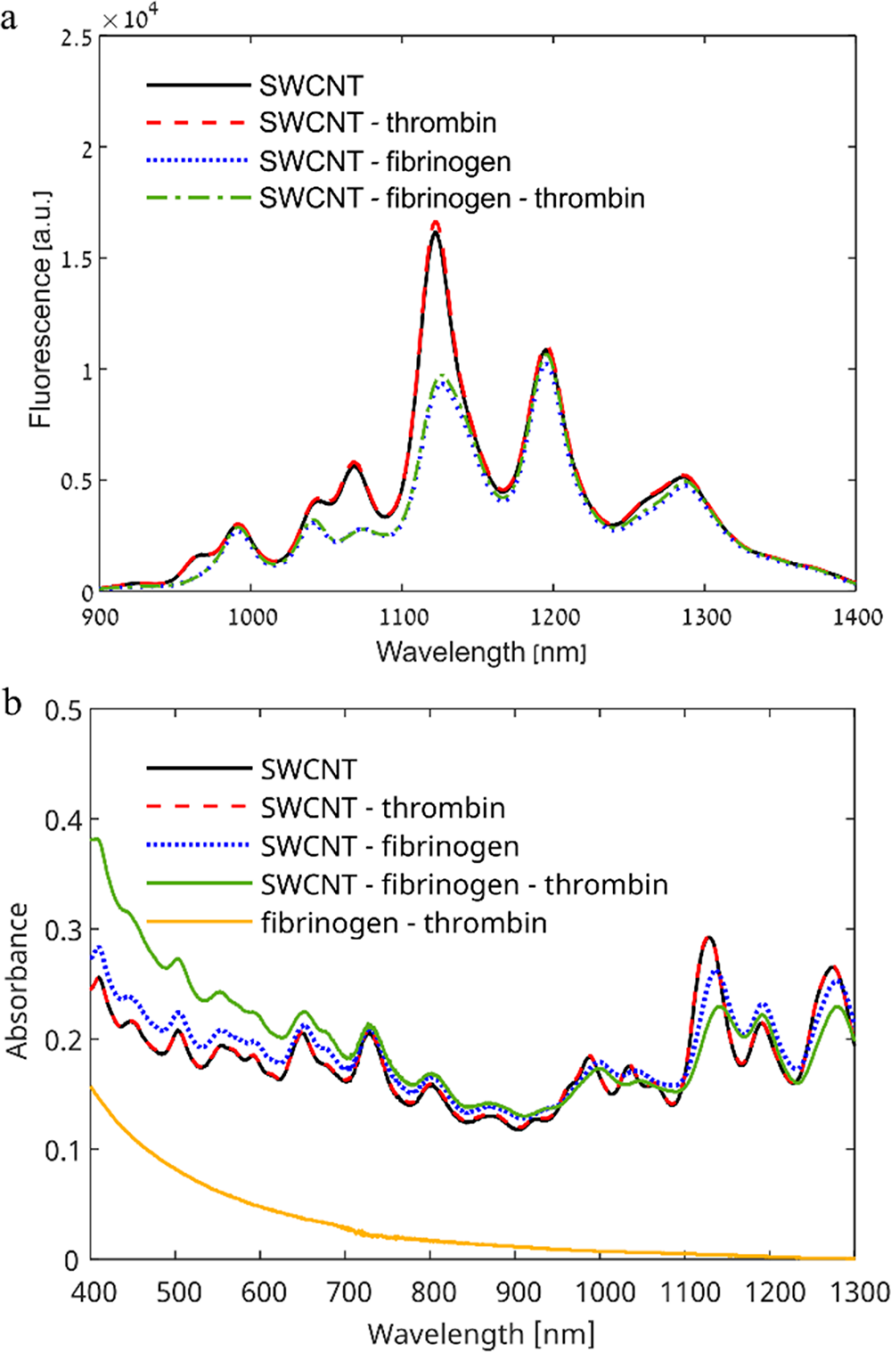
Effect of thrombin addition on DPPE–PEG–SWCNTs–fibrinogen fluorescence and absorption spectra. (a) Fluorescence emission spectra of 5 mg L^–1^ DPPE–PEG–SWCNTs without proteins (black line), with 0.02 mg mL^–1^ thrombin (dashed red line), with 0.5 mg mL^–1^ fibrinogen (dotted blue line), or with both fibrinogen and thrombin (dash-dotted green line). (b) Absorption spectra of 5 mg L^–1^ DPPE–PEG–SWCNTs without proteins (solid black line), with 0.01 mg mL^–1^ thrombin (dashed red line), with 2.5 mg mL^–1^ fibrinogen (dotted blue line), or with both fibrinogen and thrombin (solid green line) and absorption spectrum of the two proteins without DPPE–PEG–SWCNTs (solid yellow line).

We tested the effect of the addition of thrombin to the fluorescence intensity of the DPPE–PEG–SWCNTs in the presence and absence of fibrinogen. In contrast to fibrinogen, which led to a 42% fluorescence intensity decrease of the (9,4) chirality at a concentration of 0.5 mg mL^–1^ fibrinogen, the addition of thrombin did not affect the fluorescence emission of the DPPE–PEG–SWCNTs (Figure 2a). Similarly, the addition of thrombin following the addition of fibrinogen had no additional effect on the SWCNTs’ fluorescence intensity (Figure 2a). These results suggest that fibrinogen remains bound to the SWCNT surface even after the addition of thrombin and that the cleavage of fibrinopeptides converting fibrinogen to fibrin and its polymerization does not result in a modulation of the SWCNTs fluorescence. The two-step sequential binding model of fibrinogen to DPPE–PEG–SWCNTs assumed a complete binding of the three globular domains of fibrinogen to the SWCNT surface, evident from AFM imaging.^4^ The cleavage of two pairs of fibrinopeptides from the amino-terminal ends of the fibrinogen chains by thrombin^6^ exposes the fibrin polymerization site; however, it does not seem to affect the binding affinity to the SWCNTs.

We have further tested the effect of thrombin on the DPPE–PEG–SWCNTs absorption. While the addition of fibrinogen results in a slight redshift in the absorption, in agreement with previous findings,^4^ thrombin had no effect on the DPPE–PEG–SWCNTs absorption (Figure 2b). The redshift of the DPPE–PEG–SWCNTs absorption in response to fibrinogen binding was still evident even after the addition of thrombin, further supporting that the fibrinogen remained bound to the SWCNT surface. Still, there was an overall increase in the absorption in the visible wavelength range. The increase in absorption was found to be correlated to the absorption of polymerized fibrin resulting from the addition of thrombin to fibrinogen without SWCNTs (Figure S2) and was therefore attributed to the formation of fibrin clots (Figure 2b). The absorption increase was also reported in previous studies, where increased absorption was indicative of fibrin aggregation due to the formation of clots.^69^

We have further imaged the SWCNTs using transmission electron microscopy (TEM), where we could visualize individual DPPE–PEG–SWCNTs (Figure S3a) and DPPE–PEG–SWCNTs–fibrinogen (Figure S3b). TEM images of the fibrin clot without (Figure S3c) and with (Figure S3d) SWCNTs showed similar fibrillary structures. Moreover, scanning electron microscopy (SEM) images also showed similar fibrillary structures of the fibrin clot without (Figure S3e) and with (Figure S3f) SWCNTs. These high-resolution imaging results confirm that the SWCNTs did not affect the clot structure.

### 2.3 NIR-Fluorescent Imaging of SWCNTs Incorporated into Fibrin Clots

**Figure 3:**
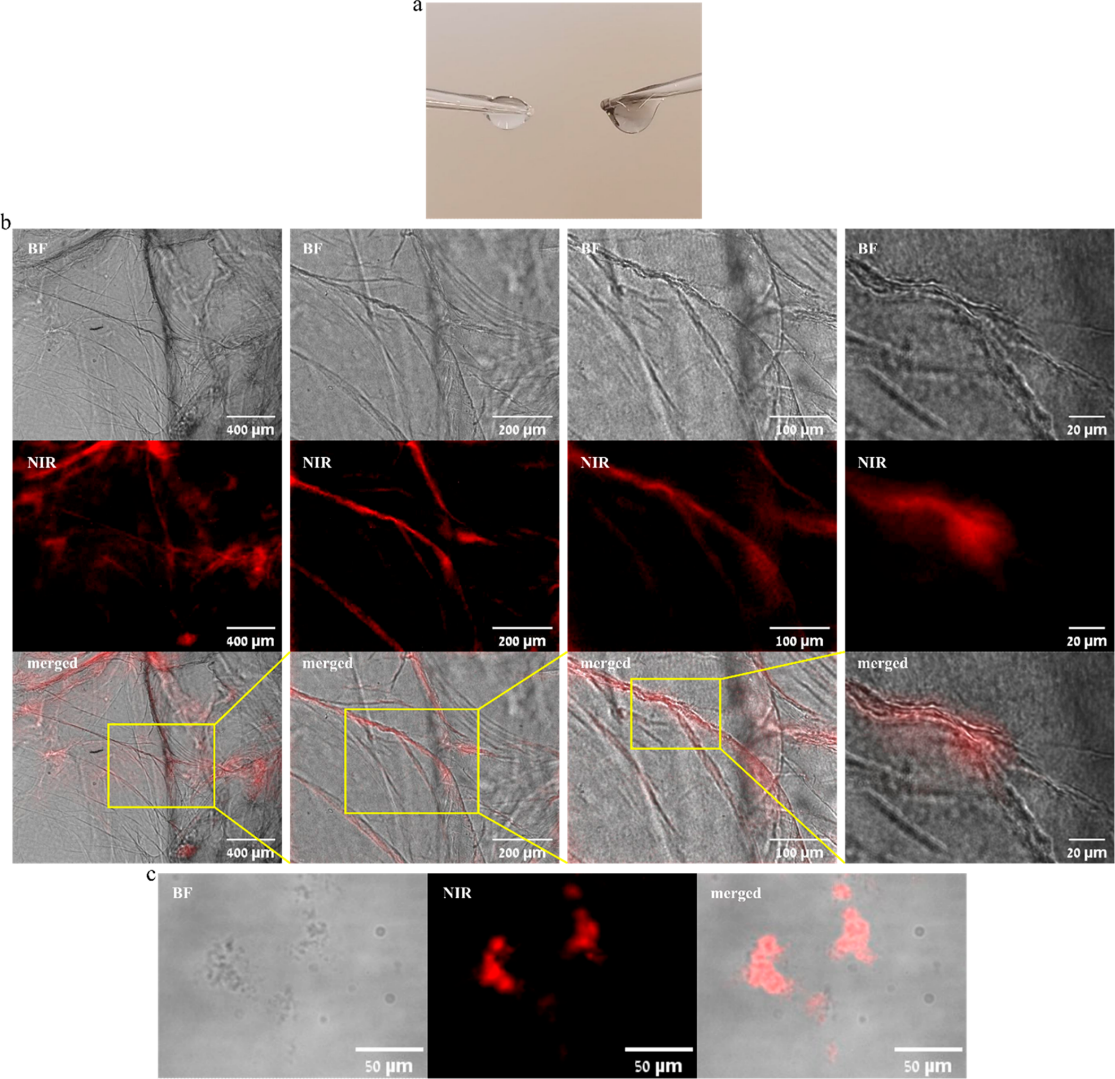
NIR-fluorescent imaging of DPPE–PEG–SWCNTs incorporated into fibrin clots. (a) Image of a fibrin clot (left) and a fibrin clot with DPPE–PEG–SWCNTs (right). (b) Bright-field (BF), NIR-fluorescent (NIR), and merged images of the DPPE–PEG–SWCNTs–fibrin clot. Images were taken with 4×, 10×, 20×, or 60× magnification (from left to right). Each image shows the region of interest marked in the image to the left. (c) Bright-field, NIR-fluorescent, and merged images of a DPPE–PEG–SWCNTs–fibrin clot in solution.

Fibrin clots consist of the assembly of fibrin monomers to fibrillary structures. We assume that SWCNTs can be integrated into these fibers, due to their thin, tubular structure. The presence of SWCNTs in the fibrin clot is supported by a simple observation of the clot with SWCNTs, which shows an opaque darker color, compared to a clot without SWCNTs, which is completely transparent (Figure 3a). To support the integration of SWCNTs into fibrin fibers, we further imaged the SWCNTs-incorporating fibrin clots using bright-field and NIR-fluorescent microscopy at different magnifications (Figure 3b) and compared these to bright-field images of fibrin clots without SWCNTs (Figure S4a). The fibrin fibers are visible in the bright field, and they show similar morphology with and without SWCNTs. Moreover, the SWCNTs clearly maintain their fluorescence emission, and we observe a colocalization between the NIR fluorescence and fibrin fibers. This correlation between the spatial distribution of the NIR fluorescence of the SWCNTs and the spatial distribution of the fibrin fibers seen in the bright-field images confirms integration of the SWCNTs into the fibers. These results ensure that the addition of thrombin does not lead to unbinding of fibrinogen from the DPPE–PEG–SWCNT surface and that SWCNTs do not hinder clot formation. In addition, we have imaged the SWCNTs-incorporating fibrin clots in 10% fetal bovine serum (FBS) and saw a similar colocalization between the SWCNTs and fibrin fibers (Figure S4b), indicating that SWCNTs–fibrin integration also occurs in a more complex environment.

Because DPPE–PEG–SWCNTs can be incorporated with the fibrin clot, they can also serve as NIR-fluorescent staining for the clots in solution. To demonstrate such staining, a DPPE–PEG–SWCNTs–fibrinogen solution was inserted into a channeled μ-slide, to which thrombin was added. After the formation of clots, both bright-field and NIR-fluorescent images were taken (Figure 3c). While in the bright-field images the clots can only be visualized in solution with very low contrast, the NIR-fluorescent images allow us to clearly identify the clots and characterize their size and location.

### 2.4 Spatiotemporal Monitoring of the Clot Formation via NIR-Fluorescent Imaging

After we established that the SWCNT can serve as NIR-fluorescent staining for the clot, we further aimed to monitor the dynamics of the clotting process. To this end, we continuously imaged the clot formation following the addition of thrombin in two different configurations, namely, in a microfluidic device under flow and on a microscope slide in a diffusion-driven process.

**Figure 4:**
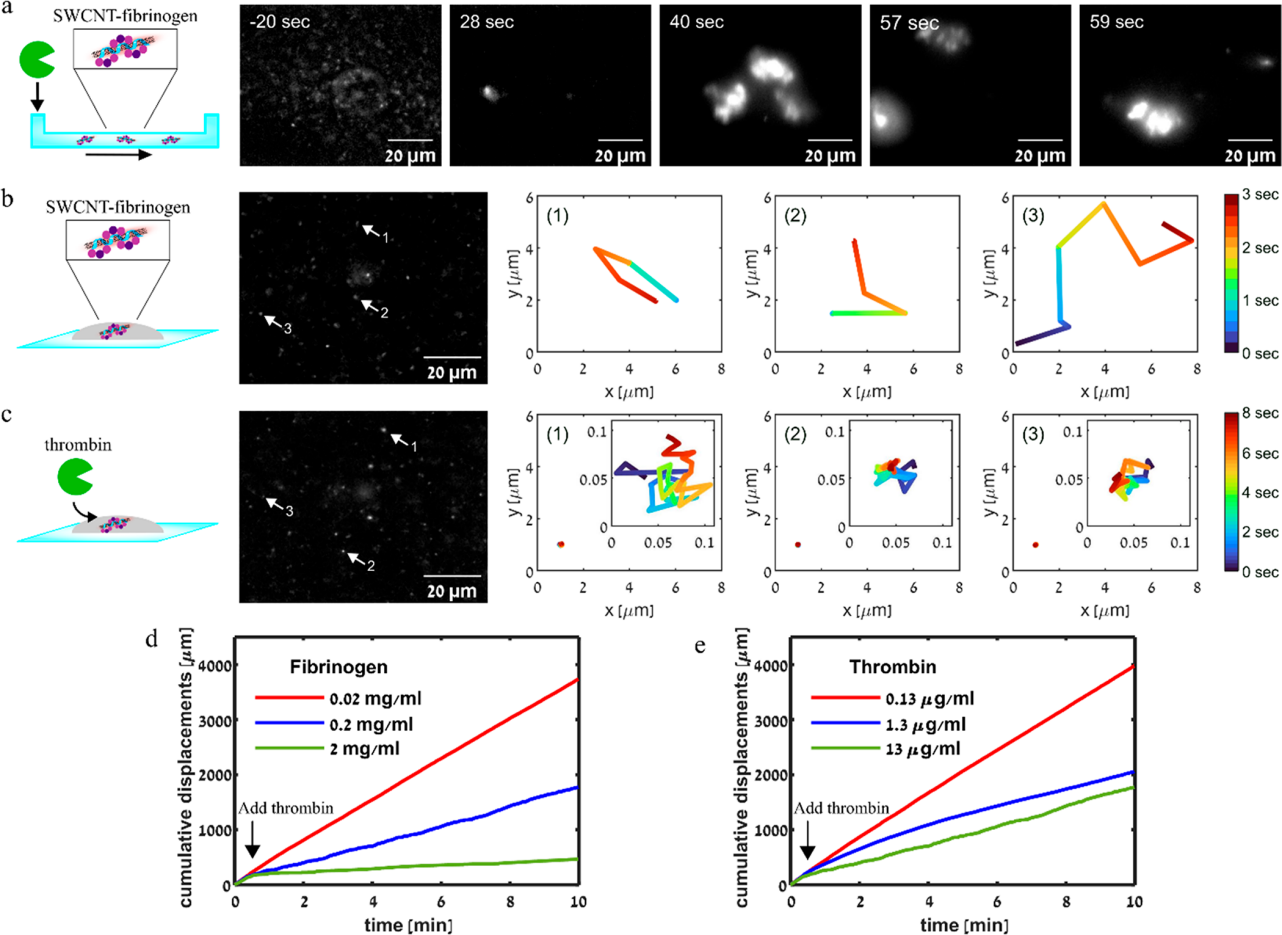
Time-resolved NIR-fluorescent imaging of the DPPE–PEG–SWCNTs–fibrinogen clotting process upon the addition of thrombin. (a) Experiments performed in a μ-slide channel. Thrombin was added at *t* = 0 s. (b) Experiments performed on a microscope slide. The temporal displacement of three individual DPPE–PEG–SWCNTs (marked with arrows) is displayed. (c) Time-resolved NIR-fluorescent imaging of DPPE–PEG–SWCNTs–fibrinogen after the addition of thrombin measured on a microscope slide. The temporal displacement of three single DPPE–PEG–SWCNTs (marked with arrows) is displayed. Inset: Same displacements over time on a large scale. (d) Cumulative displacement over time of DPPE–PEG–SWCNTs in 10% FBS incubated with different concentrations of fibrinogen—0.02 mg mL^–1^ (red), 0.2 mg mL^–1^ (blue), or 2 mg mL^–1^ (green)—before and after the addition of 13 μg mL^–1^ thrombin. (e) Cumulative displacement over time of DPPE–PEG–SWCNTs in 10% FBS incubated with 0.2 mg mL^–1^ fibrinogen before and after the addition of thrombin at different concentrations: 0.13 μg mL^–1^ thrombin (red), 1.3 μg mL^–1^ thrombin (blue), or 13 μg mL^–1^ thrombin (green).

To image the clotting process in a microfluidic channel, the SWCNTs–fibrinogen suspension was inserted into a μ-slide channel. The NIR fluorescence of the SWCNTs was imaged continuously while thrombin was added to the inlet of the channel (Supplementary Movie S1). Figure 4a shows snapshots at different time points from Supplementary Movie S1. Prior to thrombin addition, the SWCNTs appeared to be distributed throughout the solution as individual nanotubes without clusters. On the other hand, the addition of thrombin and the conversion of fibrinogen to fibrin lead to big fibrin clots of approximately 20 μm diameter. The flow, in this case, accelerates the mixing so that small clots can bind and coalesce. These clots can be visualized, due to the incorporated DPPE–PEG–SWCNTs, as high-intensity fluorescent clusters in the image.

When thrombin was added to a droplet of a SWCNTs–fibrinogen solution on a microscope slide, the clotting process was driven solely by diffusion, allowing us to follow the time-dependent diffusion behavior of the SWCNTs incorporated within the clots (Supplementary Movie S2). In contrast to the big clots that were formed under flow, we could not see large fibrin clusters in the quiescent droplet. Figure 4b shows a fluorescence image of the DPPE–PEG–SWCNTs with fibrinogen before the addition of thrombin and the displacement of three particles in the *xy* plane over several frames. The diffusion coefficient, calculated from the mean-square displacement, for these particles was 8.9 ± 7.8 μm^2^ s^–1^. After the addition of thrombin, the SWCNTs diffusion slowed down significantly, as shown in Figure 4c for the three selected particles, for which the diffusion coefficient was only 2.4 × 10^–4^ ± 8.1 × 10^–5^ μm^2^ s^–1^. We attribute these changes in the diffusion coefficients to the formation of clots around the SWCNTs, which slows down the diffusion.

Due to the significant change in the diffusion properties of the SWCNTs before and after the addition of thrombin, the NIR fluorescence can be used to monitor the fibrin clotting with both spatial and temporal resolution. We, therefore, monitored the DPPE–PEG–SWCNTs dynamics, aiming to detect changes in their diffusion when incorporated into the fibrin clots, with different concentrations of fibrinogen and thrombin. A SWCNTs–fibrinogen solution was deposited on a microscope slide and the NIR fluorescence of the SWCNTs was imaged continuously during and after the addition of thrombin in phosphate-buffered saline (PBS; Supplementary Movies S3, S4, S5, S6, and S7) and in 10% FBS (Supplementary Movies S8, S9, S10, S11, and S12). We tracked the trajectories of individual DPPE–PEG–SWCNTs during the clotting process and calculated the cumulative displacement of the particles over time. The cumulative displacement was compared for SWCNTs in different initial fibrinogen concentrations (Figures 4d and S5a) and for SWCNTs in solution with the same fibrinogen concentration upon the addition of thrombin at different concentrations (Figures 4e and S5b) in both serum (Figure 4d,e) and PBS (Figure S5a,b). In all of the experiments, we observed the same increase in the cumulative displacement as that before the addition of thrombin, indicating that the diffusion of DPPE–PEG–SWCNTs did not depend on the fibrinogen concentration in the solution. After the addition of thrombin, the cumulative displacement of DPPE–PEG–SWCNTs still increased, but with a rate that depended on the fibrinogen (Figure 4d) or thrombin (Figure 4e) concentration. A higher concentration of fibrinogen or thrombin resulted in a faster coagulation process, which was manifested in a faster decrease in the cumulative displacement rate. The concentration-dependent cumulative displacement curves can be used to formulate a calibration such that the concentration of fibrinogen or thrombin could be inferred from measured data based on a library of such curves for known concentrations. In addition, it can be used to test the final steps of the coagulation cascade where more upstream CFs are tested. Therefore, these results open new possibilities to determine the concentration of clottable fibrinogen and active thrombin and to develop new methods to monitor the coagulation cascade with real-time dynamic information, even in a complex environment.

## 3 Conclusion

We used the SWCNTs–fibrinogen sensor to monitor the formation of fibrin clots as part of the coagulation cascade. We found that the addition of thrombin did not affect the fluorescence emission of the SWCNTs or the peak absorption wavelengths, suggesting that fibrinogen remained bound to the SWCNTs surface even after the addition of thrombin and that SWCNTs did not hinder clot formation. The incorporation of SWCNTs within the fibrin clots was evident from the darker color of a macroscale clot, visible to the naked eye. We further used NIR-fluorescent imaging to visualize the SWCNTs incorporated within the fibrin clots and found that the SWCNTs were indeed integrated into the fibrillary structures of the polymerized fibrin, in both buffer and serum. Moreover, compared to the low contrast of clots in bright-field imaging in the visible range, the SWCNTs fluorescence could be used as NIR staining of the clots, for precise localization and characterization. We succeeded in monitoring the dynamics of the clotting process in two different configurations, namely, in a microfluidic channel under flow and within a droplet on a microscope slide where the process is driven by diffusion alone. In the latter case, single-particle tracking of individual SWCNTs in buffer or serum revealed different rates of clot formation depending on the concentrations of fibrinogen and thrombin, which could be used for quantification of the clottable fibrinogen and active thrombin. These results suggest that SWCNTs are a promising platform for monitoring the formation of fibrin clots and for visualizing, localizing, and tracking fibrin clots in the NIR range with both spatial and temporal resolution. Our method has the potential to improve our understanding of the blood coagulation process and could advance the development of novel diagnostic and therapeutic strategies for coagulation disorders.

## 4 Experimental Section

### 4.1 SWCNTs Suspension with DPPE–PEG

SWCNTs (HiPCO, NanoIntegris) were first suspended with 2 wt % sodium cholate (SC, Sigma-Aldrich) via bath sonication (Elma P-30H, 80 Hz for 10 min, room temperature), followed by direct tip sonication (QSonica Q125, 12 W for 30 min twice) in an ice bath. To remove SWCNT aggregates and impurities, the suspension was ultracentrifuged (OPTIMA XPN-80, 41,300 rpm for 4 h), and the top 80% of the supernatant was collected.

Subsequently, a surfactant exchange was performed. To this means, a mixture of SC-suspended SWCNTs (40 mg L^–1^) and 2 mg mL^–1^ DPPE-PEG (5000 kDa) was dialyzed against water using a dialysis cartridge (Repligen, MWCO = 1 kDa). Dialysis was performed for 5 days at room temperature with multiple water exchanges to remove SC and allow adsorption of DPPE–PEG onto the SWCNTs.

### 4.2 Absorption Spectroscopy

A successful suspension was validated by observing distinguishable peaks in the absorption spectra of the suspensions using a UV–vis–NIR spectrophotometer (Shimadzu UV-3600 PLUS), where a redshift relative to the SC-SWCNTs suspension indicated SC surfactant exchange.

The absorption spectra of a SWCNTs solution with fibrinogen and thrombin were recorded using a UV–vis–NIR spectrophotometer (Shimadzu UV-3600 PLUS). Briefly, 400 μL of 5 mg L^–1^ DPPE–PEG–SWCNTs was measured with 0 or 2.5 mg mL^–1^ fibrinogen and 0 or 0.01 mg mL^–1^ thrombin in PBS. The absorption was recorded between 400 and 1300 nm (1 nm step size).

### 4.3 NIR-Fluorescent Spectroscopy

Fluorescence emission spectra were recorded in a 96-well plate mounted on an inverted microscope (Olympus IX73). A supercontinuum white-light laser (NKT-photonics, Super-K Extreme) with a band-width filter (NKT-photonics, Super-K Varia, Δλ = 20 nm) was coupled to a microscope as the excitation source. Fluorescence emission was spectrally resolved using a spectrograph (Spectra Pro HRS-300, Teledyne Princeton Instruments) with a slit width of 500 μm and a grating (150 g mm^–1^). The fluorescence intensity spectrum was recorded by an InGaAs detector (PyloNIR, Teledyne Princeton Instruments).

For validation of a successful suspension, the fluorescence spectra of 100 μL of a 1 mg L^–1^ SC–SWCNTs or DPPE–PEG–SWCNTs suspension were recorded at an excitation wavelength of 730 nm (20 mW) with an exposure time of 1 s. A redshift in the DPPE–PEG–SWCNTs fluorescence spectrum relative to the SC–SWCNTs suspension indicated SC surfactant exchange.

For the fluorescence spectra of DPPE–PEG–SWCNTs with fibrinogen and thrombin, 100 μL of a 1 mg L^–1^ SWCNT suspension was incubated with fibrinogen for 1 h. Then, thrombin was added and incubated for 20 min. The fluorescence spectra were recorded at an excitation wavelength of 730 nm (20 mW) with an exposure time of 1 s.

To quantify the DPPE–PEG–SWCNTs–fibrinogen interaction, the fluorescence peak intensity of the (9,4) chirality was determined, and its concentration-dependent response on the addition of fibrinogen was fitted with a two-step sequential binding model of the DPPE–PEG–SWCNTs–fibrinogen interaction. The data were fitted by the equation^4^
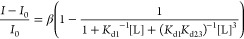
1where *I*_0_ is the initial fluorescence intensity, *I* is the final fluorescence intensity, [*L*] is the fibrinogen concentration, β is a proportionality factor, and *K*_d1_ and *K*_d23_ are the dissociation constants. The resulting fit parameters were β = −0.59, *K*_d1_ = 0.0115 mg mL^–1^, and *K*_d23_ = 2.21 × 10^–5^ mg^2^ mL^–2^.

Excitation–emission maps were recorded on samples of 1 mg L^–1^ DPPE–PEG–SWCNTs in PBS with 0.5 mg mL^–1^ fibrinogen or without fibrinogen using an excitation wavelength range of 500–840 nm in 2 nm steps with an exposure time of 1 s.

### 4.4 TEM

A total of 30 μL of the sample solutions: 5 mg L^–1^ DPPE–PEG–SWCNTs, 5 mg L^–1^ DPPE–PEG–SWCNTs with 5 mg mL^–1^ fibrinogen, a fibrin clot from 5 mg mL^–1^ fibrinogen and 5 μg mL^–1^ thrombin, and a similar concentration of fibrin clot with 5 mg L^–1^ DPPE–PEG–SWCNTs (in PBS, final concentration) were applied to a carbon-coated grid and stained with 10 μL of a 2% (w/v) uranyl acetate solution. After blotting with an excess stain solution, the grid was left to air-dry. The negatively stained samples were imaged using a JEM-1400Plus transmission electron microcsope (JEOL, Japan). Images were captured using a SIS Megaview III camera and iTEM as the TEM imaging platform (Olympus).

### 4.5 SEM

A total of 40 μL of the sample solutions: a fibrin clot from 5 mg mL^–1^ fibrinogen and 5 μg mL^–1^ thrombin and a similar concentration of fibrin clot with 5 mg L^–1^ DPPE–PEG–SWCNTs (in PBS, final concentration) were dried under vacuum and coated with 5 nm gold particles. The samples were imaged using a high-resolution scanning electron microscope (Zeiss GeminiSEM 300) with an EDS detector (X-Flash 6/60, Bruker).

### 4.6 2D Fluorescence Imaging

Fluorescence images were taken via an inverted fluorescence microscope (Olympus IX83) through a 100X TIRF objective (Olympus UAPON 100XOTIRF). The SWCNTs fluorescence was excited by a 730 nm continuous-wave laser (MDL-MD-730-1.5W, Changchun New Industries). The laser excitation light was directed to the samples with a dichroic mirror (900-nm-long pass, Chroma), and the NIR emission of the SWCNTs was detected after an additional 900-nm-long-pass emission filter (Chroma, ET900lp) with an InGaAs camera (Raptor, Ninox 640 vis–NIR). All images were processed by *ImageJ* and *MATLAB*.

For the SWCNTs–fibrin clot images, 1 mg L^–1^ DPPE–PEG–SWCNTs, 2 mg mL^–1^ fibrinogen, and 0.002 mg mL^–1^ thrombin in PBS or in 10% FBS were mixed, and the SWCNTs–fibrin clot was transferred from the solution to a microscope slide.

### 4.7 Measurements of DPPE–PEG–SWCNTs during Fibrin-Clot Formation

For images of the coagulation process measured in flow, a mixture of 4 mg L^–1^ DPPE–PEG–SWCNTs and 2 mg mL^–1^ fibrinogen was inserted into a channel slide (Ibidi). The fluorescence images were taken at time intervals of 200 ms with an exposure time of 50 ms. After 13 s, while the images were still taken, 20 μL of 0.1 mg mL^–1^ thrombin was added to the inlet of the channel. After formation of the clots, both bright-field and NIR images were taken.

For images of the coagulation process measured on the slide, a mixture of 20 μL of 1 mg L^–1^ DPPE–PEG–SWCNTs in PBS or in 10% FBS and 2, 0.2, or 0.02 mg mL^–1^ fibrinogen was dropped on a microscope slide. The fluorescence images were taken at time intervals of 200 ms with an exposure time of 150 ms. After a few seconds, while the images were still taken, 10 μL of thrombin was added to obtain final concentrations of 13, 1.3, or 0.13 μg mL^–1^ of the SWCNTs–fibrinogen solution on the microscope slide.

SWCNTs displacement was calculated using the *TrackMate* toolbox in *ImageJ*.^70^ To calculate the cumulative increments, the mean displacement of the particles between two consecutive frames of the movie was calculated. The cumulative increments in each time are the sum of the mean displacement until the corresponding frame.

## Supporting Information Available

Normalized fluorescence spectra of SC–SWCNTs and DPPE–PEG–SWCNTs suspensions, absorption spectra of fibrinogen–thrombin with and without SWCNTs, TEM and SEM images of SWCNTs and fibrin clots, bright-field images of fibrin clots without SWCNTs and bright-field and NIR images of SWCNTs–fibrin clot in 10% FBS, and cumulative displacement over time of DPPE–PEG–SWCNTs incubated with different concentrations of fibrinogen and thrombin in PBS (PDF)
Movie S1: SWCNT_2 fibrinogen_0.1 thrombin_channel_2 min.avi (AVI)
Movie S2: SWCNT_2 fibrinogen_13 thrombin_40 s.avi (AVI)
Movie S3: SWCNT_0.02 fibrinogen_13 thrombin_10 min.avi (AVI)
Movie S4: SWCNT_0.2 fibrinogen_13 thrombin_10 min.avi (AVI)
Movie S5: SWCNT_2 fibrinogen_13 thrombin_10 min.avi (AVI)
Movie S6: SWCNT_0.2 fibrinogen_0.13 thrombin_10 min.avi (AVI)
Movie S7: SWCNT_0.2 fibrinogen_1.3 thrombin_10 min.avi (AVI)
Movie S8: SWCNT_FBS _0.02 fibrinogen_13 thrombin_10 min.avi (AVI)
Movie S9: SWCNT_FBS _0.2 fibrinogen_13 thrombin_10 min.avi (AVI)
Movie S10: SWCNT_FBS _2 fibrinogen_13 thrombin_10 min.avi (AVI)
Movie S11: SWCNT_FBS _0.2 fibrinogen_0.13 thrombin_10 min.avi (AVI)
Movie S12: SWCNT_FBS _0.2 fibrinogen_1.3 thrombin_10 min.avi (AVI)

## Supplementary Material

am3c00828_si_001.pdf

am3c00828_si_002.avi

am3c00828_si_003.avi

am3c00828_si_004.avi

am3c00828_si_005.avi

am3c00828_si_006.avi

am3c00828_si_007.avi

am3c00828_si_008.avi

am3c00828_si_009.avi

am3c00828_si_010.avi

am3c00828_si_011.avi

am3c00828_si_012.avi

am3c00828_si_013.avi
