# Supplementary material for: Monitoring the Formation of Fibrin Clots as Part of the Coagulation Cascade Using Fluorescent Single-Walled Carbon Nanotubes
Source: ACS Appl Mater Interfaces. 2023 May 2;15(18):21866–76. doi: 10.1021/acsami.3c00828 (PMC10176323; doi:10.1021/acsami.3c00828)
Supplement: Supplementary file 1 — am3c00828_si_001.pdf [file am3c00828_si_001.pdf]

Supporting Information

Monitoring the formation of fibrin clots as part of the coagulation cascade using fluorescent single-walled carbon nanotube

Efrat Gerstman[a], Adi Hendler-Neumark[a], Verena Wulf[a], Gili Bisker[a,b,c,d,]*

[a]*Department of Biomedical Engineering, Faculty of Engineering, Tel Aviv University, Tel Aviv 6997801, Israel*

[b]*Center for Physics and Chemistry of Living Systems, Tel Aviv University, Tel Aviv 6997801, Israel*

[c]*Center for Nanoscience and Nanotechnology, Tel Aviv University, Tel Aviv 6997801, Israel*

[d]*Center for Light-Matter Interaction, Tel Aviv University, Tel Aviv 6997801, Israel*

E-mail: bisker@tauex.tau.ac.il

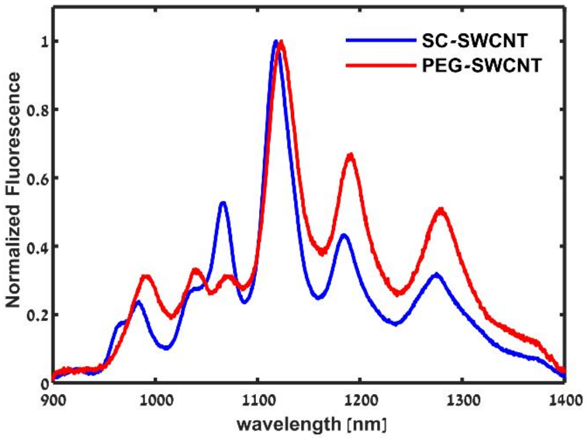

Figure S1. Normalized fluorescence spectra of SC-SWCNT suspension (blue) and the red-shifted DPPE-PEG-SWCNT suspension (red), indicating a successful exchange of SC by DPPE-PEG on the SWCNT surface.

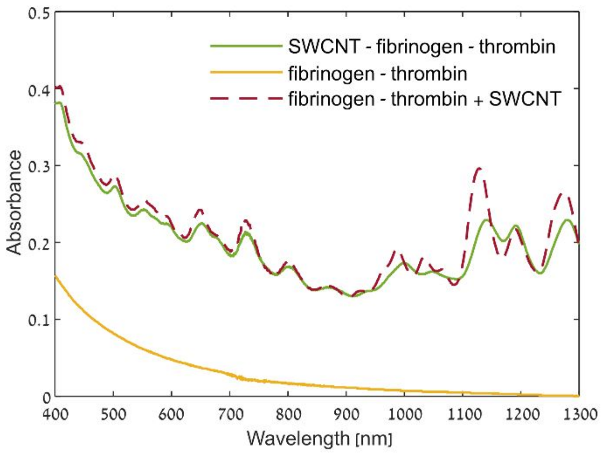

Figure S2. The effect of DPPE-PEG-SWCNT addition on absorption spectra of fibrin clots. Absorption spectrum of 5 mg L^{-1} DPPE-PEG-SWCNT with both 2.5 mg mL^{-1} fibrinogen and 0.01 mg mL^{-1} thrombin (SWCNT – fibrinogen – thrombin, green line), absorption spectrum of the two proteins, 2.5 mg mL^{-1} fibrinogen and 0.01 mg mL^{-1} thrombin, without DPPE-PEG-SWCNT (fibrinogen – thrombin, yellow line), and the absorption spectrum of the two proteins without DPPE-PEG-SWCNT to which the absorption of DPPE-PEG-SWCNT was added (fibrinogen – thrombin + SWCNT, dashed brown line). The similar absorption spectra of SWCNT – fibrinogen – thrombin and fibrinogen – thrombin + SWCNT indicate that the increase in absorption of the SWCNT – fibrinogen – thrombin sample compare to DPPE-PEG-SWCNT alone, stems from the formation of the clot, and that the presence of SWCNT does not hinder the clotting process.

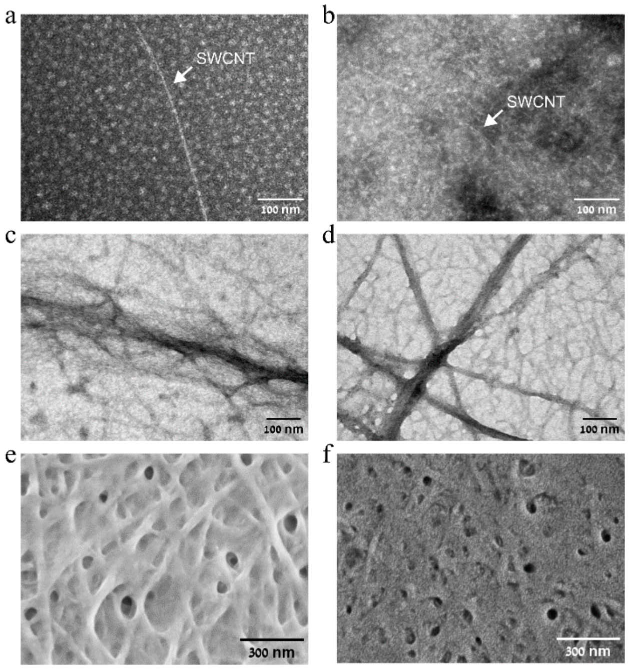

Figure S3. The effect of DPPE-PEG-SWCNT addition on the structure of the fibrin clot. a) TEM image of DPPE-PEG-SWCNT. b) TEM image of DPPE-PEG-SWCNT-fibrinogen. c) TEM image of a fibrin clot. d) TEM image of fibrin clot with SWCNT. e) SEM image of a fibrin clot. f) SEM image of fibrin clot with SWCNT. The similar fibrillary structures of the fibrin clot with or without SWCNT confirm that the SWCNT has no effect on the clot structure.

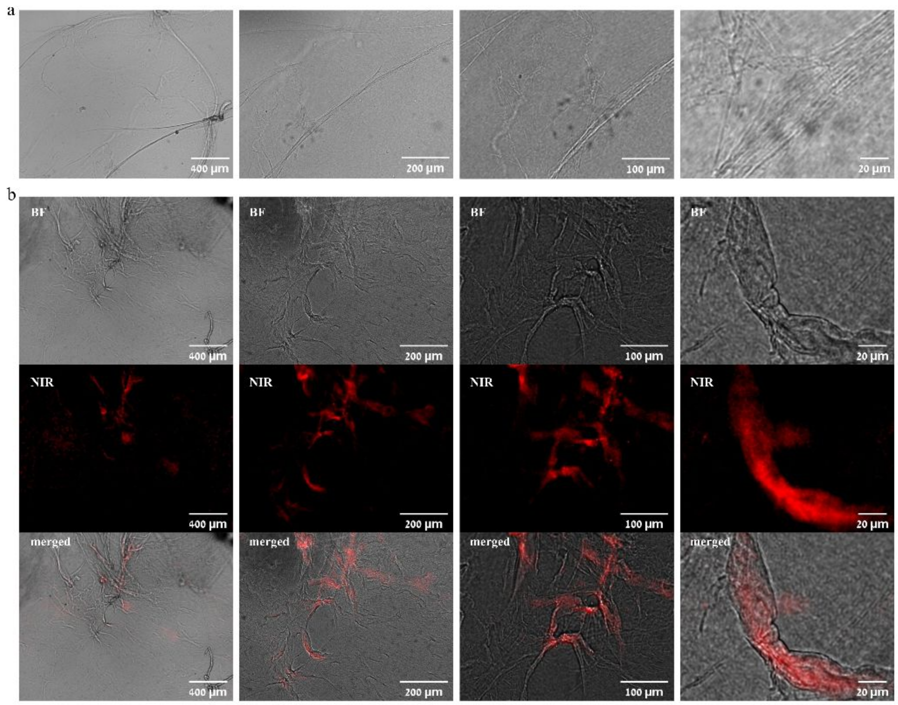

Figure S4. a) Brightfield images of fibrin clots in PBS without DPPE-PEG-SWCNTs. Images were taken with 4x, 10x, 20x, or 60x magnification (from left to right). b) NIR-fluorescence imaging of DPPE-PEG-SWCNTs incorporated into fibrin clots in 10% Fetal bovine serum (FBS). Brightfield (BF), NIR-fluorescence (NIR), and merged images of the DPPE-PEG-SWCNT-fibrin clot. Images were taken with 4x, 10x, 20x, or 60x magnification (from left to right).

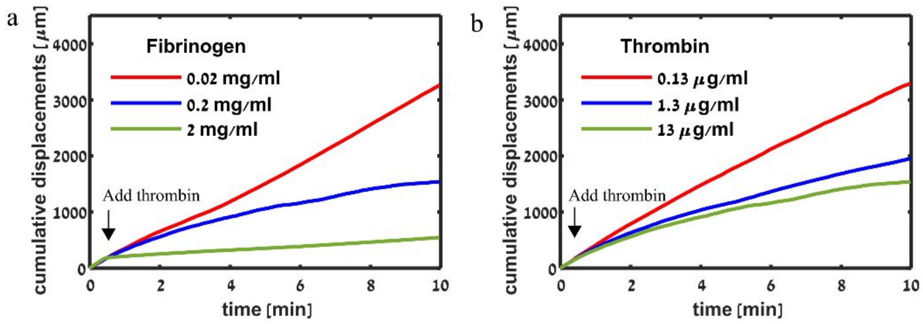

Figure S5. Time-resolved NIR-fluorescence imaging of DPPE-PEG-SWCNT-fibrinogen clotting process upon the addition of thrombin in PBS. a) Cumulative displacement over time of DPPE-PEG-SWCNT incubated with different concentrations of fibrinogen: 0.02 mg mL^{-1} (red), 0.2 mg mL^{-1} (blue), or 2 mg mL^{-1} (green) before and after the addition of 13 µg mL^{-1} thrombin. b) Cumulative displacement over time of DPPE-PEG-SWCNT incubated with 0.2 mg mL^{-1} fibrinogen before and after the addition of thrombin at different concentrations: 0.13 µg mL^{-1} thrombin (red), 1.3 µg mL^{-1} thrombin (blue), or 13 µg mL^{-1} thrombin (green).

SUPPLEMENTARY MOVIES:

Movie S1: SWCNT_2 fibrinogen_0.1 thrombin_channel_2 min.avi

Time-lapse video of NIR fluorescence of DPPE-PEG-SWCNT – fibrinogen in a channel slide. 20 μl of 0.1 mg mL^{-1} thrombin was added to the channel inlet at $t = 13$ sec. The movie was taken with 100X magnification at 5 frames per second (FPS) and exposure time of 50 ms.

Movie S2: SWCNT_2 fibrinogen_13 thrombin_40 sec.avi

Time-lapse video of NIR fluorescence of DPPE-PEG-SWCNT – fibrinogen on a microscope slide. 13 μg mL^{-1} thrombin (final concentration) was added at $t = 25$ sec. The movie was taken with a 100X magnification at 5 FPS and exposure time of 150 ms.

Movie S3: SWCNT_0.02 fibrinogen_13 thrombin_10 min.avi

Movie S4: SWCNT_0.2 fibrinogen_13 thrombin_10 min.avi

Movie S5: SWCNT_2 fibrinogen_13 thrombin_10 min.avi

Movie S6: SWCNT_0.2 fibrinogen_0.13 thrombin_10 min.avi

Movie S7: SWCNT_0.2 fibrinogen_1.3 thrombin_10 min.avi

Time-lapse videos of NIR fluorescence of DPPE-PEG-SWCNT – fibrinogen on a microscope slide with fibrinogen concentration of 0.02 (S3), 0.2 (S4, S6, and S7), or 2 (S5) mg mL^{-1}. 0.13 (S6), 1.3 (S7), or 13 (S3, S4, and S5) μg mL^{-1} thrombin (final concentrations) was added at the $t = 23$ sec. The movies were taken with 100X magnification at 5 FPS and exposure time of 150 msec.

Movie S8: SWCNT_FBS_0.02 fibrinogen_13 thrombin_10 min.avi

Movie S9: SWCNT_FBS _0.2 fibrinogen_13 thrombin_10 min.avi

Movie S10: SWCNT_FBS _2 fibrinogen_13 thrombin_10 min.avi

Movie S11: SWCNT_FBS _0.2 fibrinogen_0.13 thrombin_10 min.avi

Movie S12: SWCNT_FBS _0.2 fibrinogen_1.3 thrombin_10 min.avi

Time-lapse videos of NIR fluorescence of DPPE-PEG-SWCNT – fibrinogen in 10% FBS on a microscope slide with fibrinogen concentration of 0.02 (S3), 0.2 (S4, S6, and S7), or 2 (S5) mg mL^{-1}. 0.13 (S6), 1.3 (S7), or 13 (S3, S4, and S5) μg mL^{-1} thrombin (final concentrations) was

added at the $t = 23$ sec. The movies were taken with 100X magnification at 5 FPS and exposure time of 150 msec.
